# A Chance-Constrained Vehicle Routing Problem for Wet Waste Collection and Transportation Considering Carbon Emissions

**DOI:** 10.3390/ijerph17020458

**Published:** 2020-01-10

**Authors:** Hailin Wu, Fengming Tao, Qingqing Qiao, Mengjun Zhang

**Affiliations:** 1 College of Mechanical Engineering, Chongqing University, Chongqing 400044, China; wu_hailin@foxmail.com (H.W.); qiaoqingqing@cqu.edu.cn (Q.Q.); zhangmengjun@cqu.edu.cn (M.Z.); 2 School of Management Science and Real Estate, Chongqing University, Chongqing 400044, China

**Keywords:** wet waste collection and transportation, chance-constrained programming, carbon emissions, smart waste bins

## Abstract

In order to solve the optimization problem of wet waste collection and transportation in Chinese cities, this paper constructs a chance-constrained low-carbon vehicle routing problem (CCLCVRP) model in waste management system and applies certain algorithms to solve the model. Considering the environmental protection point of view, the CCLCVRP model combines carbon emission costs with traditional waste management costs under the scenario of application of smart bins. Taking into the uncertainty of the waste generation rate, chance-constrained programming is applied to transform the uncertain model to a certain one. The initial optimal solution of this model is obtained by a proposed hybrid algorithm, that is, particle swarm optimization (PSO); and then the further optimized solution is obtained by simulated annealing (SA) algorithm, due to its global optimization capability. The effectiveness of PSOSA algorithm is verified by the classic database in a capacitated vehicle routing problem (CVRP). What’s more, a case of waste collection and transportation is applied in the model for acquiring reliable conclusions, and the application of the model is tested by setting different waste fill levels (WFLs) and credibility levels. The results show that total costs rise with the increase of credibility level reflecting dispatcher’s risk preference; the WFL value range between 0.65 and 0.75 can obtain the optimal solution under different credibility levels. Finally, according to these results, some constructive proposals are propounded for the government and the logistics organization dealing with waste collection and transportation.

## 1. Introduction

Solid waste management (SWM) has always been the most concerned issue in every region [1,2] which is composed of many stages including generation, collection and transportation, treatment and disposal [3,4]. The process of collection and transportation is one of the most challenging steps among all aspects of SWM [5]. Specially in developing countries, up to 80–90% of municipal budgets is reserved for waste collection and transportation services [6] which is identified as the most expensive functional element in SWM [1], while the frequency and efficiency are still very low [6]. Therefore, waste collection route optimization is the principal component for achieving the best savings in SWM [7]. In this paper, we consider the collection and transportation of wet waste (defined as biodegradable waste [8]), a kind of solid waste, which is placed in specific waste bins in or around residential areas, then collected and transported to the disposal center by special waste trucks.

Road transportation is the most common mode used for wet waste transportation, during which fuel is consumed and carbon dioxide equivalent emissions are produced. With the emergence of environmental pollution problems, the issue of carbon emissions has attracted much attention [9,10]. What is more, vehicles produce emissions not only when driving, but also when loading and unloading waste due to the necessity to keep their engines running, producing constant exhaust emissions [11,12]. These considerations highlight the importance of optimizing vehicle routing to reduce the carbon emissions during the process of waste collection and transportation [13].

At present, most research on waste collection is carried out considering fixed routes and collection of all the waste bins according to a predetermined schedule, but this method is not particularly good. In particular, the use of fixed routes might lead to half-full waste bins, overflowing waste bins and high fuel consumption, which are very serious problems [9]. For these reasons, many cities and regions are starting to use smart waste bins to reduce operating costs and improve residents’ satisfaction through real-time monitoring of waste volumes. Generally, a smart waste bin is a kind of waste collector which is equipped with different technical devices, such as different sensors systems and RFID to monitor the waste level of bin and achieve the communication between smart waste bins and trucks [5,14,15], accordingly allowing the implementation of real-time optimized transportation routes instead of fixed routes [13].

In short, it is necessary to study both of the wet waste generation rate and carbon emissions in the process of wet waste collection and transportation. Therefore, we must consider the following questions: How to plan the vehicle routes under the scenario of application of smart waste bins; how to handle the stochastic variable of waste generation rate; how to quantify the carbon emissions of waste collection vehicles; and how to build a comprehensive optimization model that considers costs and carbon emissions. Thus, this paper is organized as follows: a literature review of related work is presented in Section 2. The model formulation is proposed in Section 3. The proposed algorithm is described in Section 4. The algorithm experiment and model experiment are shown in Section 5. Finally, conclusions are presented in Section 6.

## 2. Literature Review

The main idea of this article is about waste collection and transportation considering the waste generation rate which will be handled by chance-constrained programming against the background of smart waste bins’ application. In the model, the carbon emissions are also considered. We review the literature in three areas: waste collection and transportation (vehicle routing problems, carbon emissions and smart waste bins), chance-constrained programming, and heuristic algorithms.

### 2.1. Research about Waste Collection and Transportation

There is a series of studies about waste collection and transportation including all kinds of waste. Kim et al. [16] established a VRPTW model for commercial waste collection, taking into account multiple waste treatments and driver rest time. Zsigraiova et al. [12] established a vehicle route optimization model for glass waste collection based on a GIS system. Asefi et al. [17] considered different types of waste carried by different vehicles, established a vehicle routing model with the lowest total cost and workload balance of different transfer stations. Markov et al. [18] established a recyclable waste collection routes optimization problem considering random inventory and established a waste prediction model based on sensor data and historical data of the waste collection container.

A range of techniques has been employed in an optimization model for SWM with diverse focus and objectives [19], for example, smart waste bins. With the development of science and technology, smart waste bins are gradually being introduced. Akhtar et al. [5] established a waste collection model considering the application of smart bins. The research results show that the optimal threshold waste level of waste bin is between 70% and 75%, and the improved model and algorithm perform better in path optimization. Some researches apply smart waste bins in real cases, including glass bins in Geneva, Switzerland [18] and residential waste bins in the UAE [20]. Maurizio et al. [11] regarded the amount of waste generated as a random variable. Real-time data were acquired through modern traceable devices such as RFID, GPRS and GPS, and corresponding rules are set to determine whether waste bins should be collected.

Vehicles are the main mode of waste transportation, which can release a lot of carbon dioxide. Thus, some scholars have studied the carbon emissions in the process. Jabbarzadeh et al. [21] established a model to solve the optimal transportation route with the objective of minimizing greenhouse gas emissions cost in the waste management system. Herold and Lee [22] took 40 global logistics companies as examples and discussed how the extent of the dynamic interaction between internal and external practices influences carbon disclosure strategies. Herold et al. [23] illustrated how the interaction of institutional and stakeholder pressures influences a company’s carbon disclosure and depicted four types of carbon disclosure strategies. Edalatpour et al. [24] estimated the average social cost of carbon and the amount of carbon dioxide produced per ton of dry and wet waste. Then sustainable benefits from reducing carbon dioxide emissions are calculated and taken as the benefit component in the objective function.

We can see from the above research that many kinds of waste are considered for collection and transportation from the environmental point of view. However, wet waste which is collected separately has been rarely studied in the implementation of waste classification considering carbon emissions both of driving and idling which is important for the waste collection vehicles. The technology of smart waste bins is widely studied, yet the application of smart waste bins in the stage of waste collection and transportation is rarely considered.

### 2.2. Research about Chance-Constrained Programming

When we consider the waste generation rate, we will be confronted with a stochastic number; therefore, we will need a stochastic programming method to solve the model [24]. The chance-constrained programming technique was introduced by Charnes and Cooper for stochastic programming [25] and has been applied to solve the various kinds of VRPs considering stochastic variables.

Edalatpour et al. [24] established and solved a generic waste management model considering the waste generation rate by a chance-constraint method and analyzed the influence of significant levels on the objective function. Zhang et al. [26] developed a multi-echelon supply model in which the waste generation rate is a stochastic variable, and is transformed into a deterministic constraint. Xu et al. [27] constructed a model combining a genetic algorithm and fuzzy chance-constrained programming to support SWM under uncertainty conditions, and the applicability of the proposed model was demonstrated by a real reginal waste management issue. Men et al. [28] considered a HazMat equipped vehicle routing problem (H-CVRP) in a type-2 fuzzy environment. Because of the stochastic variable of population density, the objective function involved trapezoidal interval type-1 variables, and was transformed into two equivalent constrains by chance-constrained programming. Kundu et al. [29] proposed a solid transportation problem with fuzzy variables including availability, demands and conveyance capacities, and chance-constrained programming was employed to solved the problem. Kundu et al. [30] investigated multi-objective solid transportation problems considering various uncertainties, including stochastic penalties, fuzzy resource, demands, conveyance capacities and budget. In the model, the uncertain contains are reformulated into deterministic ones by a chance-constrained programing technique.

From the above studies, we can see that chance-constrained programming is effective for stochastic variables, but few articles have applied it to waste generation rates in the field of waste collection and transportation.

### 2.3. Research about Some Algorithms for Waste Collection and Transportation

There are various methods for VRP including metaheuristic, exact method, classic heuristic, real-time solution and simulation [31]. The VRP problem is a NP-hard problem. Therefore, it is natural to use a heuristic to solve the problem [32], such as the adaptive Harmony Search Algorithm (HSA) [33], adaptive Large Neighborhood Search (LNS) algorithm [34], Iterated Greedy Algorithm (IGA) [35], Genetic Algorithm (GA) [3] and Particle Swarm Optimization (PSO) algorithm [36], Simulated Annealing Algorithm (SAA) [28,37,38], Multiple Neighborhood Search (MNS) algorithm [24], etc.

Waste collection and transportation is one type of application of VRP, and many studies have solved the VRP in the context of MSW. Jacobsen [39] formulized a municipal solid waste (MSW) collection and transportation problem into a mixed integer program and a heuristic solution was proposed for the problem. Buhrkal et al. [32] studied the waste collection vehicle routing problem with time windows considering worker lunch breaks. The greedy algorithm was employed to construct the initial solution which was improved by the adaptive large neighborhood search with the destroy and repair methods and the results illustrated the usefulness of the algorithm. Hemmelmayr et al. [40] considered a waste collection node routing problem, then the variable neighborhood search and insertion were done by dynamic programming. Schneider et al. [41] was the first person to introduce the vehicle routing problem with intermediate stops (VRPIS). To solve the complex problem, the author combined the strong diversification of varying neighborhood search with an adaptive mechanism to get a highly efficient heuristic, characterized by short computing times and high-quality results.

In summary, the studies about algorithms for waste collection and transportation have appeared extensively but most of them used a single algorithm, rather than a hybrid algorithm which can learn from others’ strong points and close the gap. In view of this, the paper proposed a model considering carbon emissions and waste generation rate which will be handled by chance-constrained programming for wet waste collection and transportation.

## 3. Model Formulation

### 3.1. Problem Description

There is a wet waste disposal center used as the depot with a certain number of wet waste collection vehicles and a set of smart waste bins to be collected. The location of the disposal center and each smart waste bin are known. The wet waste volume and quantity can be monitored. The waste generation rate during the period from collection vehicle’s departure to smart waste bins is a stochastic parameter and the chance constraint programming is applied to deal with this uncertainty Additionally, the carbon emissions are considered both in driving and idling. The goal of the problem is to find an optimal solution considering the factor of cost, waste generation rate and environment. The detailed assumptions in this paper are as follows:

(1) There is only one type of wet waste collection vehicles with limited capacity.
(2) The location of the depot and each smart waste bin are known.
(3) Each waste generation point has its independent stochastic rate of waste generation and obeys normal distribution [24].
(4) All waste collection vehicles must return the depot when collection tasks completed.
(5) Each smart waste bin is only collected by one vehicle once.

### 3.2. Notation

Based on the needs to establish the model, Table 1 presents the corresponding notation applied in this paper.

### 3.3. Model Construction

The model of waste collection and transportation in this paper takes vehicle cost including the fixed vehicle cost and the variable vehicle cost and the carbon emissions as the objective functions. Above all, we analyze the components of three objective functions separately, and then the specific formulation of the model is determined by these components.

#### 3.3.1. Analysis of Objectives Function

1. Vehicle cost

(1) Fixed vehicle cost

Fixed cost refers to the capital cost, insurance cost, tax cost and salvage cost and etc., the total fixed cost $C_{f}$ in the model can be calculate as:

(1) $$C_{f}=\overset{K}{\underset{k=1}{\sum }}\overset{n}{\underset{j=0}{\sum }}x_{0jk}p_{f}$$

(2) Variable vehicle cost

Variable cost refers to the cost of fuel from two activities including driving and idling. Driving means the activity between the collection nodes, idling means the fuel consumption of loading on a certain bin and unload waste. What is more, the distance and load determine the driving fuel, and the idling fuel consumption is dependent on the time of idling [42].

The paper refers to literature [43] to calculate the fuel cost. From the analysis above, we can know fuel consumption rate (FCR) of driving is the linear function of vehicle load, thus, when the load is $Q_{1}$, FCR can be calculated with the equation:

(2) $$\rho \left(Q_{1}\right)=\rho_{0}+\frac{\rho^{*}-\rho_{0}}{Q}Q_{1}$$

In the CCLCVRP model, FCR of driving can be expressed as:

(3) $$\rho_{ij}=\rho_{0}+\frac{\rho^{*}-\rho_{0}}{Q}\overset{i-1}{\underset{m=0}{\sum }}x_{mlk}\left(q_{m}+\Delta q_{m}\right)$$

(a) Total fuel cost of driving is calculated as:

(4) $$C_{fuel-drive}=\overset{N}{\underset{i=0}{\sum }}\overset{N}{\underset{j=0}{\sum }}x_{ijk}d_{ij}\rho_{ij}p$$

(b) Total fuel cost of idling can be expressed as:

(5) $$C_{fuel-idel}=\overset{N}{\underset{j=1}{\sum }}x_{ijk}t_{j}\rho_{idel}p$$

(c) Total variable vehicle can be calculated by the below equation:

(6) $$C_{v}=C_{fuel-drive}+C_{fuel-idel}$$

2. Carbon Emissions Cost

The literature has shown that the carbon emissions of vehicles have a relationship with fuel consumption [44]. The paper uses the following Equation (7) to calculate the amount of carbon dioxide emissions from fuel consumption:

(7) $$E_{CO_{2}}=\eta F_{fuel}$$

The total amount of fuel consumption is decided by the distance of driving and the time of idling:

(8) $$F_{fuel}=\overset{N}{\underset{i=0}{\sum }}\overset{N}{\underset{j=0}{\sum }}x_{ijk}d_{ij}\rho_{ij}+\overset{N}{\underset{j=1}{\sum }}x_{ijk}t_{j}\rho_{idel}$$

(9) $$E_{CO_{2}}=\eta \left(\overset{N}{\underset{i=0}{\sum }}\overset{N}{\underset{j=0}{\sum }}x_{ijk}d_{ij}\rho_{ij}+\overset{N}{\underset{j=1}{\sum }}x_{ijk}t_{j}\rho_{idel}\right)$$

Total carbon emission cost can be calculated as:

(10) $$C_{c}=\varepsilon E_{CO_{2}}$$

#### 3.3.2. Model Setting

On the basis of the above analysis, the mathematical model built in the study is as follows:

(11) $$MinF=\overset{K}{\underset{k=1}{\sum }}\overset{n}{\underset{j=0}{\sum }}x_{0jk}p_{f}+\overset{N}{\underset{i=0}{\sum }}\overset{N}{\underset{j=0}{\sum }}x_{ijk}d_{ij}\rho_{ij}p+\overset{N}{\underset{j=1}{\sum }}x_{ijk}t_{j}\rho_{idel}p\;+\varepsilon \eta \left(\overset{N}{\underset{i=0}{\sum }}\overset{N}{\underset{j=0}{\sum }}x_{ijk}d_{ij}\rho_{ij}+\overset{N}{\underset{j=1}{\sum }}x_{ijk}t_{j}\rho_{idel}\right)$$

Subject to:

(12) $$\overset{K}{\underset{k=1}{\sum }}\overset{N}{\underset{i=0}{\sum }}x_{ijk}=1,\;\forall j=1,2,\cdots ,N$$

(13) $$\overset{K}{\underset{k=1}{\sum }}\overset{N}{\underset{j=0}{\sum }}x_{ijk}=1,\;\forall i=1,2,\cdots ,N$$

(14) $$\overset{N}{\underset{i=0}{\sum }}x_{ijk}=\overset{N}{\underset{i=0}{\sum }}x_{jik}=1,\;\forall j=1,2\cdots ,N;k=1,2,\cdots ,K$$

(15) $$\overset{N}{\underset{j=1}{\sum }}x_{ijk}\left(q_{j}+\Delta q_{j}\right)\leq Q,\;i=0,1,\cdots ,N;k=1,2,\cdots ,K$$

(16) $$\overset{N}{\underset{i=0}{\sum }}\overset{N}{\underset{j=0}{\sum }}d_{ij}x_{ijk}\leq L,\;\forall k=1,2,\cdots ,K,\;i\neq j$$

(17) $$\overset{N}{\underset{i=0}{\sum }}\overset{N}{\underset{j=0}{\sum }}x_{ijk}\leq \left|S\right|-1,\;S\subseteq \left\{1,2,\cdots ,N\right\},\;\forall k=1,2,\cdots ,K$$

The objective function (11) is to minimize the total cost including vehicle cost and carbon emission cost. Constraint (12) shows that each waste bin must be collected once by a vehicle. A vehicle begins at the depot and ends at the last visited customer, which is imposed by Constraint (13) and (14). Constraint (15) represents that the amount of bins for each path cannot larger than the maximum load of the vehicle. The total length for each route does not exceed the longest length of route, which is provided by Constraint (16). Constraint (17) illustrates the subtour elimination.

### 3.4. Chance-Constrained Programming

As an efficient tool for decision making in uncertain environments to hedge risk, chance-constrained programming (CCP) has brought more attention recently. In a CCP problem, decision makers are interested in satisfying a constraint by at least a pre-specified probability at the minimum cost [45]:

(18) minψ(x)s.t. P{C(x,ξ)}≥1−γx∈X

where x∈X represents the decision variables, ψ often represents a convex cost function, ξ represents a random vector defined on a probability space and the set function P{} represents the probability distribution.

Due to the greatly uncertain of waste generation rate [46], it’s difficult to predict the amount of generated waste during the period from collection vehicle’s departure to smart waste bins, which is set as a stochastic variable in constraint (15). Thus, in order to solve the model, it necessary to convert the uncertain constraint to a certain one by CCP. For the stochastic variable Δq in constraint (15) of the CCLCVRP model, the CCP mode is stated as follows:

(19) $$\mathrm{Pr}\left[\overset{N}{\underset{j=1}{\sum }}x_{ijk}\left(q_{j}+\Delta q_{j}\right)\leq Q\right]\geq \gamma ,\;i=0,1,\cdots ,N;k=1,2,\cdots ,K$$

where γ is the predefined credibility level;

According to $\Delta q_{i}\sim N\left(\mu_{i},\sigma_{i}^{2}\right)$, we have the following equivalent constraints:

(20) $$\overset{N}{\underset{j=1}{\sum }}x_{ijk}\mu_{j}+\Phi^{-1}\left(\gamma \right)\sqrt{\overset{N}{\underset{j=1}{\sum }}{\left(x_{ijk}\right)}^{2}\sigma_{j}^{2}}+\overset{N}{\underset{j=1}{\sum }}x_{ijk}q_{j}\leq Q$$

So far, constraint (15), the stochastic part of the model is transformed into the determined one by chance-constrained programming.

## 4. Algorithm

### 4.1. Algorithm Design

**Figure 1:**
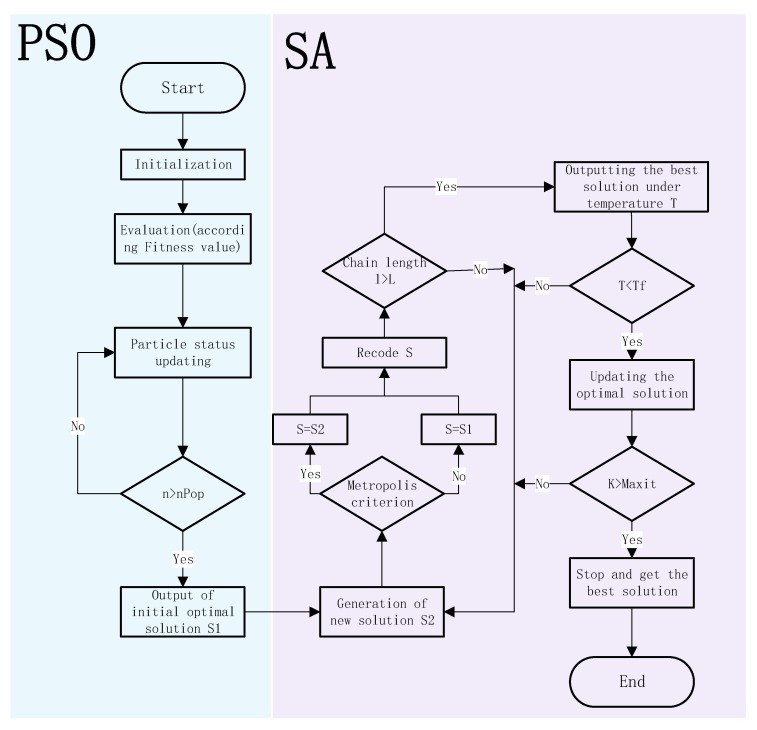
The Flowchart of PSOSA.

Because of the NP-hard [47] nature of the proposed problem, which combines CVRP with stochastic variables, exact methods need lots of time to solve large sized problems optimally. Therefore, meta-heuristic algorithms were developed to achieve optimal or near optimal solutions in a reasonable time for large sized problem instances. The meta-heuristic algorithms try to generate some random solutions initially. Then by searching in the neighborhoods of better solutions, they try to find near optimal solutions. Meta-heuristic algorithms are divided into two categories: solution-based and population-based algorithms. Since population-based algorithms have more speed and accuracy in finding optimal solutions [36], a Particle Swarm Optimization (PSO) algorithm is adopted to generated an initial optimal solution. Considered that the problem with PSO is premature convergence and local minima. To avoid this, better exploration is required and hence PSO is equipped with Simulated Annealing (SA) algorithm. SA is a local search-based algorithm that has a mechanism to escape from local optimum with the purpose of finding a global optimum. The PSOSA algorithm detailed flow chart for solving the CCPLCVRP model is shown in Figure 1.

### 4.2. Encoding and Decoding

There is a major problem with the PSO algorithm, which is how the position of the particle corresponds to the solution of the model. The encoding and decoding of this particle can ensure that each smart waste bin is served once and that each smart waste bin can be limited to only one vehicle. What is more, the calculation of the solution process can be reduced. This article constructs a coding method for the particle, according to literature [44]. All particles are composed of three parts: Part 1 has B particles (B is the number of smart waste bins), the value of each particle represents the vehicle number as well as the sub-path number to which each smart waste bin belongs and is randomly selected from the natural number of 1 to V; Part 2 has B particles (from 1 to B) which is the sequence number of smart waste bins; Part 3 has B particles(randomly generated between 0–1), the size of the value decides the order of the various smart waste bin in each sub-path.

For example, there is a depot (number 0), 10 smart waste bins (number 1 to 10) and three vehicles (1 to 3). For the following particles:

As displayed in Table 2, the first line is about part1 includes 1, 2 and 3, indicating that there are three vehicles and three sub-paths. Part 2 is showed in the second line from 1 to 10 representing the smart waste bin sequence. It can be seen the smart waste bins of 3, 4, 9 and 10 are allocated to the sub-path1; 2, 5and 7 are allocated to the sub-path2; 1, 6 and 8 are allocated to the sub-path3. Part 3 corresponding to the third line of Table 2 decides the visiting order of each sub-path and to make it clear the fourth line gives the number order of the third line. Each vehicle starts from the depot and back to the depot after finishing their assignment. Thus, the sub-path1 is 0-4-10-3-9-0. The second sub-path is 0-7-2-5-0. The third sub-path is 0-8-6-1-0.

### 4.3. Constructing Initial Optimal Solution Based on PSO Algorithm

First, we use the PSO algorithm to obtain a high-quality initial solution. When the algorithm starts, each solution in the solution space is considered as a particle. In the shrinking space, each particle has a position to determine its position and a speed to determine its distance and direction.

#### 4.3.1. Initialization and Fitness Evaluation

Parameter initialization [44], sets the length of particle code VarSize, the number of population nPop, maximum number of iterations MaxIt, the number of $r_{1}$, $r_{2}$, acceleration factor $c_{1}$, $c_{2}$, and the particle range [VarMin, VarMax] and velocity range [−0.1×(VarMax−VarMin), 0.1×(VarMax−VarMin)]. The solution vector is a 1-dimensional variable in this paper. When the particles are initialized, the position and velocity of the *i-*th population can be expressed as:

(21) $$x_{i}=rand\left(VarSize\right).\times \left(VarMax-VarMin\right)+VarMin$$

(22) $$v_{i}=rand\left(VarSize\right).\times \left(VelMax-VelMin\right)+VarMin$$

All particles have a fitness value to evaluate the strength that is determined by the fitness function, which is calculated based on the cost of each route.

#### 4.3.2. Determining Optimal Solution

In each iteration, each particle has an individual extremum, which is expressed as $p^{best}$ and all particles share a global extremum, which is expressed as $g^{best}$. Particles follow the individual extremes and global extremes to search in the solution space and find the optimal solution.

#### 4.3.3. Particle Status Update

Each time the position and velocity are updated according to Equation (23):

(23) $$\{\begin{array}{c} v_{i+1}=\omega_{i+1}v_{i}+c_{1}r_{1}\left(p^{best}-x_{i}\right)+c_{2}r_{2}\left(g^{best}-x_{i}\right)\\ If\;v_{i+1}>v_{max},\;v_{i+1}=v_{max}\\ If\;v_{i+1}<v_{min},\;v_{i+1}=v_{min}\\ x_{i+1}=x_{i}+v_{i+1} \end{array}$$

Generally, to improve the convergence of the particles, inertia weight (ω) is used. Since the search space is complex, a time variant inertia weight is used which is specified in Equation (24):

(24) $$\omega_{i+1}=\omega_{i}\times wdamp$$

In Equation (24) wdamp is the inertia weight damping ratio, which is between 0–1. Initially, a high inertia weight is used leading to high global exploration. As the iteration progresses, the inertia weight is lowered to guide local exploitation.

#### 4.3.4. Terminating Condition

Finally, when the greatest population number nPop appears is the end of the condition. Otherwise, it will continue to evolve.

### 4.4. Structure Global Optimal Solution Based on SA

This paper combines annealing algorithm with the particle swarm optimization algorithm in order to make for the shortcoming of poor local search ability of the particle swarm optimization algorithm and produce a high quality solution.

#### 4.4.1. Initialization and Initial Solution

Parameter initialization, set the initial temperature $T_{0}$, the final temperature $T_{end}$, the chain length for each temperature L. The optimal solution obtained by PSO algorithm is set as the initial solution of SA algorithm.

#### 4.4.2. Generation of New Solution

If the new solution is not as good as the initial solution, it is not necessarily discarded. Thus, Metropolis criterion is invoked to determine whether to accept a worse solution or not which is described as Equation (25):

(25) $$p=\{\begin{array}{c} \exp\left(-\frac{\Delta f}{T_{i}}\right),\Delta f\geq 0\\ 1,\Delta f<0 \end{array}$$

New solution is newsolu, the objective function value is newobjv, and Δf is used to represent the increment of the objective function, Δf=newobj−objv. $T_{i}$ is the current temperature. If newobjv is less than obj, the new solution is unconditionally accepted. If newobjv is higher than obj, the probability of acceptance of the new solution is $p=\exp\left(-\frac{\Delta f}{T_{i}}\right)$.

For each temperature, a series of L attempts are performed to explore the space and the best solution under each temperature is recorded.

#### 4.4.3. Cooling Operation

During the process, the temperature decreases by multiplying the cooling coefficient r to enforce the convergence as is shown by Equation (26):

(26) $$T_{i+1}=T_{i}\times r$$

#### 4.4.4. Terminating Condition

Finally, that the temperature $T_{i}$ is lower than the final temperature $T_{end}$ is the end of the condition. Otherwise, it will continue to evolve.

## 5. Numerical Experiments and Analysis

### 5.1. Algorithm Experiment

In this section,10 cases (A-n32-k5, A-n36-k5, A-n46-k7, A-n53-k7, A-n62-k8, A-n80-k10, B-n51-k7, F-n135-k7, P-n76-k5 and P-n101-k4) are chosen from the typical database in CVRP to test the applicability of design PSOSA algorithm. Table 3 shows the information about the test instances which includes the number of nodes and the capacity of vehicles. The related parameters are set according to the literature [37,48,49], as shown in Table 4. In this study, the traditional PSO algorithm is compared with the proposed PSOSA algorithm. Each of the following experiments is executed 20 times with Matlab R2016b on a PC with Intel core i5 CPU operating at 2.60 GHz. The best value is recorded as the optimal results. Table 5 shows the detailed computational results of PSO and PSOSA.

**Figure 2:**
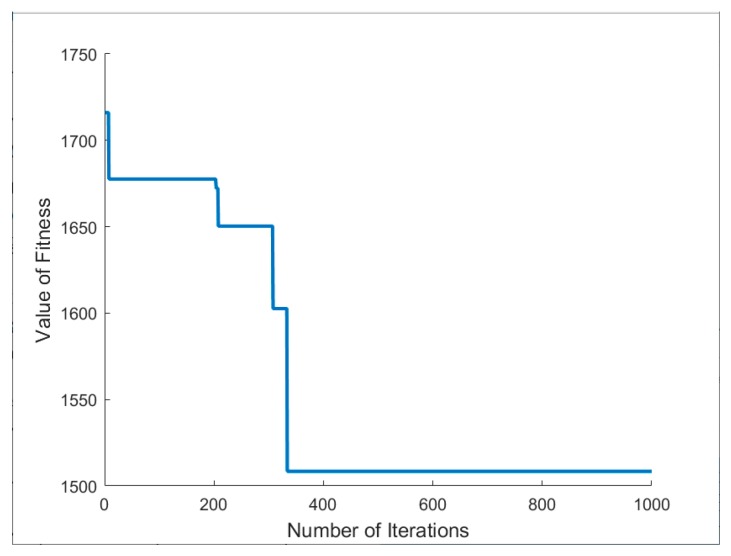
Convergence results of PSOSA algorithm.

Figure 2 shows some values of the iterative process of PSOSA algorithm. It can be seen in Figure 2 that the highest fitness value has been obtained when the number of iterations of the PSOSA algorithm is less than 500. We can easily see from Table 5 that, compared with the PSO algorithm, the results obtained by PSOSA including the total cost, the carbon emissions and the route length are better. Overall, they have a great improvement in the quality of the solution. Thus, the proposed PSOSA algorithm in this paper is effective and competitive in tackling VRP.

### 5.2. Model Experiment

#### 5.2.1. Experimental Design

The distribution data of a vehicle routing problem concerning waste collection and transportation, which is referred from [50], is used to verify the CCLCVRP model. There is one wet waste disposal center used as the depot and several waste collection vehicles of the same kind with a capacity of 2000 kg. There are 30 smart waste bins to be collected. Considering that the study is aimed at wet waste, the waste amount in original data is processed by multiplied by 60%.

Detailed information is displayed in Table 6, including of the position and amount of waste. And we set the other parameters of the CCLCVRP model according to the former studies [44,51,52], which are shown in Table 7.

#### 5.2.2. Experimental Results

1. Experimental Results of Comparison between CCP and EVM

CCP is an efficient tool to solve the stochastic problem and credibility level γ in CCP is predetermined representing the probability of path success without overloading and reflecting the dispatcher’s risk preference. The higher the γ, the lower the risk. Firstly, the comparison experiment with Expected Value Method (EVM) is designed to verify the rationality of CCP. EVM refers to the incremental amount of waste is predicted according to the average values without considering disturbance factor [53]. In order to evaluate the solution of the two methods more effectively, the robustness [53] of the solution is defined, as is stated in Equation (27):

(27) $$Robustness=\mathrm{Pr}\left[\overset{N}{\underset{j=1}{\sum }}x_{ijk}\left(q_{j}+\Delta q_{j}\right)\leq Q\right]$$

The robustness of the solution indicates the probability of path success, that is, the probability of not overloading. The EVM and CCP with different γ are implemented 20 times and the robustness is shown in Table 8.

We can see from Table 8, EVM has a lower robustness, while the CCP has a higher one which is close to the predefined credibility levels (γ).

2. Experimental Results of WFLs and Credibility Levels

Different combinations of WFLs and credibility levels will result in different optimal solutions. Thus, secondly, we do the following experiments to achieve optimal optimization. Each experiment is implemented 20 times, and the numerical value with the best result is recorded.

In order to evaluate the solution more comprehensively, two concepts are introduced, tightness and unit cost. Tightness [54] is estimated by calculating the amount of waste carried per unit vehicle capacity, as shown in Equation (28). Unit cost is about the collection cost of per unit waste and is stated in Equation (29):

(28) $$Tightness=\frac{Total\;collected\;waste}{NO.of\;vehicles\times Capacity\;of\;vehicles}$$

(29) $$Unit\;Cost=\frac{Total\;cost}{Total\;collected\;waste}$$

Then we do two initial experiments: (1) an experiment about WFLs in a static environment, that is, no consideration of γ; (2) an experiment about γ, that is, WFL = 0. Finally, we do a sensitivity analysis of WFLs under different credibility levels.

(i) Experimental Results of WFLs

**Figure 3:**
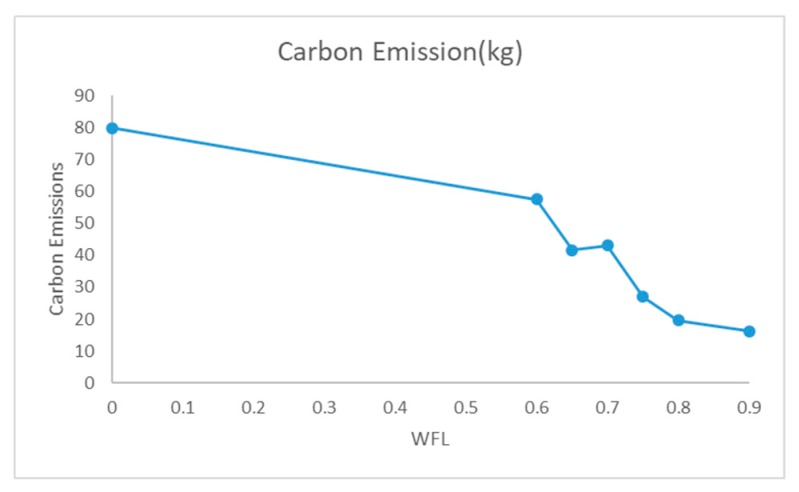
Caron Emissions at Different WFLs.

**Figure 4:**
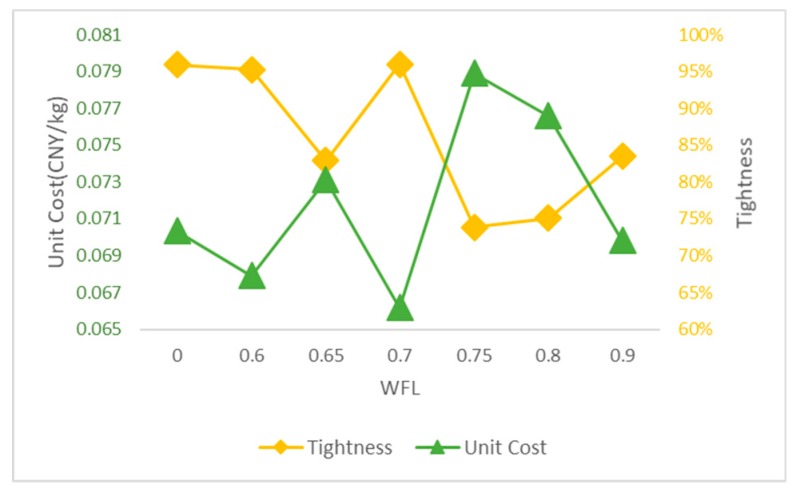
Changes of Tightness and Efficiency at Diffident WFLs.

We consider seven WFLs, namely, 0, 0.6, 0.65, 0.7, 0.75, 0.8 and 0.9 [54], to compute an efficient waste collection route, and waste bin exceeding a certain WFL needs to be collected. Table 9 shows the obtained results, including total cost (C), carbon emissions (CE), total length (L), improvement (I), the number of collected smart waste bins (n), the number of vehicles used (N), detail route (Route), total collected waste (TCW), the percentage of total collected waste ($TCW_{p}$), tightness (T) and unit cost (UC).The applying of WFL concept shows impressive results on smart waste bin efficiency compared with the conventional pattern (WFL = 0). The model showed improved results when using the smart waste bin. With the increase of WFL, the total cost, carbon emissions and total length decrease and the fifth column shows the improvement in total cost under different WFLs compared with conventional pattern. Obviously, the increase in WFL leads to the drop both in the number of collected smart waste bins and the number of vehicles. To illustrate the impact of smart waste bins on environment, Figure 3 shows the carbon emissions at different WFLs. We can see carbon emission reached a highest value without the use of smart waste bins (WFL = 0) and carbon emissions decrease, as WFL increases. To better explain the change in tightness and efficiency, the results are shown in Figure 4. Generally, the less unit cost means the better savings of cost and carbon emissions, and the higher tightness means the good savings of distance. Figure 4 shows that we can get the good value both of tightness and unit cost at the WFL of 0.7 for the database.

(ii) Experimental Results of Credibility Levels

The amount of waste generated within the waste generation points are not always determined over time, so we realistically consider the waste generation rate as a stochastic parameter. This means that when collection vehicles depart from the depot according to the schedule and the amount of waste in smart waste bins, there will be an incremental of waste amount in smart waste bins as travelling time elapses, so a chance constraint method is applied to deal with this uncertainty and credibility levels are predefined to insure the probability of routes’ success. The means and standard deviations of the incremental for each bin are random assigned values, respectively.

**Figure 5:**
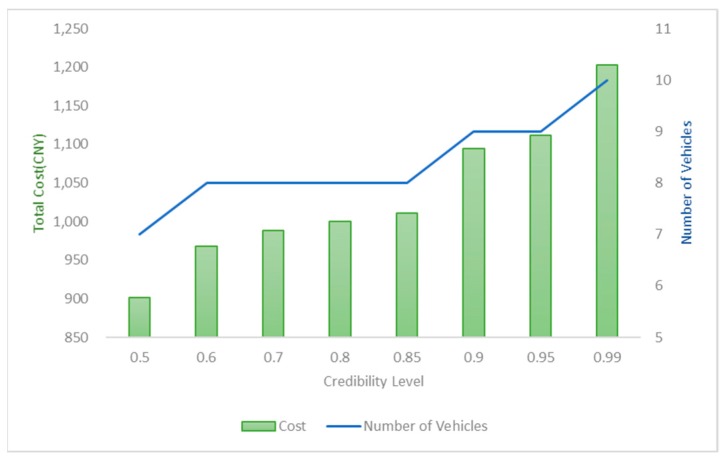
Total Cost and Number of Vehicles under Different Credibility Level γ.

We do the sensitivity analysis under the condition of WFL = 0 (all the smart waste bins will be collected according to the schedule). Obviously, the objective function varies with the predefined credibility levels and the objective values of the same solution are different with different credibility levels. As shown in Figure 5, the total cost of this optimal solution increase and the number of vehicles does not reduce as γ increases. It is worth mentioned that for γ = [0.5,0.6], [0.85,0.9] and [0.95,0.99], the total cost of optimal solution has a sharp increase with the number of vehicles grows. For other point, there is a slight change. The predetermined γ reflects the dispatchers’ different attitudes toward risk [55]. dispatchers can accept the risk brought by path failure with a lower γ; otherwise, dispatchers hope the actual amount of waste will be less than the expected one with a high γ to avoid overloading.

3. Experiment results of WFLs under different γ

**Figure 6:**
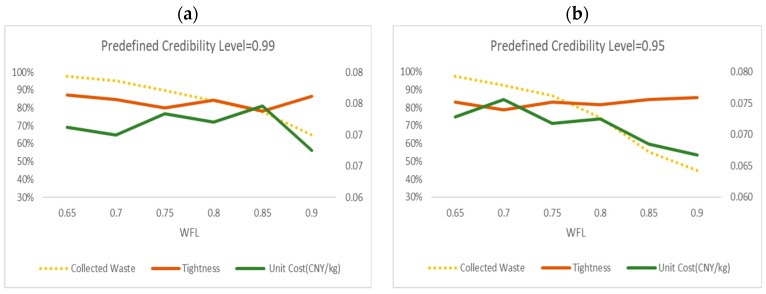

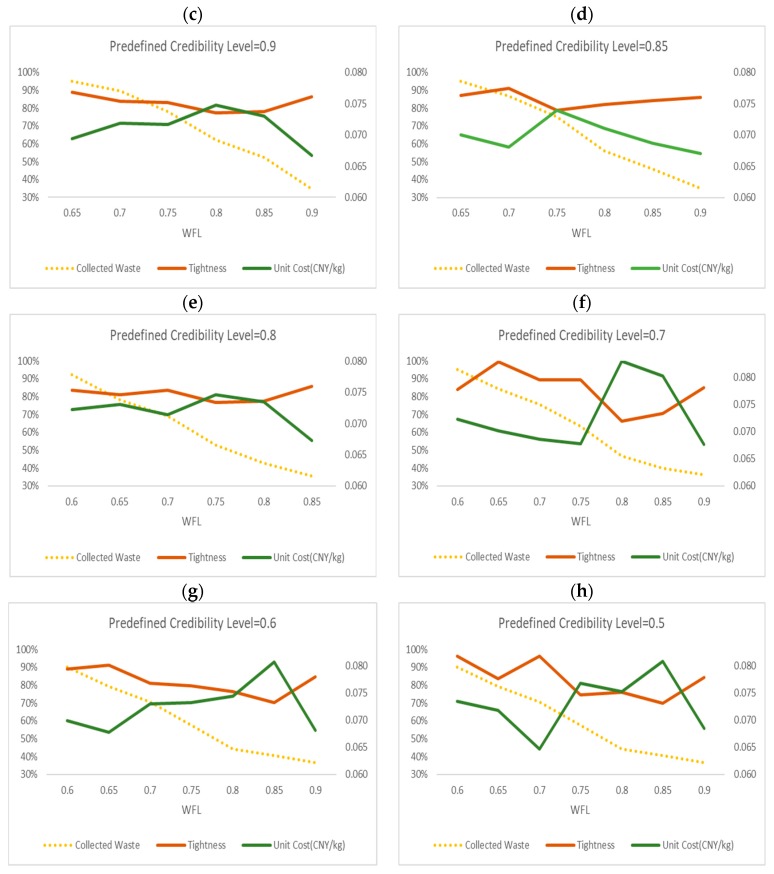
The changes of tightness, unit cost and collected waste percentage under different *γ*.

This section deals with the improvement in waste collection and route optimization by implying smart waste bins under different credibility levels. As shown in Figure 6, we adopt the different predefined credibility levels γ of 0.99, 0.95, 0.9, 0.85, 0.8, 0.7, 0.6 and 0.5 to obtain the tightness and unit cost of the optimal solutions under different WFLs. The results illustrate that both of the higher tightness above 80% and lower cost about 0.07 CNY/kg are generated at 0.65 or 0.75 of WFL. If 0.9 of WFL is considered, the high efficiency can be realized, however, the collected waste percentage is less than 60% under all the predefined credibility levels, which will be inconvenient for waste collection vendors. Taking Figure 6d for example, we can see the better solution can be obtained at the WFL of 0.7 with 91% tightness, 0.068 CNY/kg of unit cost and 87% collected waste percentage under the credibility level of 0.85. Although the efficiency is not bad at WFL of 0.9, the total collected waste percentage is about 35%, which is too little to leading to overflowing for some smart waste bins. Nevertheless, for different credibility levels, the set of WFL at 0.65 or 0.75 provides the most efficient and optimized values.

### 5.3. Analysis of Results

For the VRP in waste management, the CCLCVRP model is built in this paper aiming at the optimization of the wet waste collection and transportation system. Under the application of smart waste bins, we study the impact of different WFLs and credibility levels on the total cost, unit cost, tightness and the percentage of collected waste. Obviously, it is difficult to determine a certain optimal value of WFL under different credibility levels, because it will change as various factors change, including the waste management decision, waste generation rate and so on. However, we can confirm a value range of WFL to achieve the overall optimality of the waste collection and transportation system with the application of smart waste bins. In this paper, by assigning different numerical combination of WFLs and credibility levels, we obtain the optimal solutions by PSOSA. The main summings-up are listed as follows:

(1) For experiment 1, when the WFL is increasing, all of the total cost, carbon emissions and route length decrease, and the unit cost and tightness fluctuate. In the static environment, there is a certain WFL of 0.75 with the minimum unit cost and the highest tightness.
(2) For experiment 2, when γ∈[0.8, 1), the dispatcher is the risk aversion type, expecting to plan more vehicles to obtain higher path reliability; when γ∈[0.5, 0.8), the manager is the risk preference type, thinking that the overloading risk caused by uncertain environment can be accepted.
(3) For experiment 3, in the range between 0.65 and 0.75 of WFL, the waste transportation obtains the overall optimality under different credibility levels.
(4) Through setting different value of WFLs and credibility levels, it is proved that the CCLCVRP model is applicable for waste collection and transportation.

Based on the above results, some constructive suggestions are put forward. From the perspective of waste collection and transportation organizations, they can apply scientific ways such as path optimization methods and technical means such as smart waste bins to reduce the total cost. In a static environment, the WFL of 0.75 is a good choice to ensure the efficiency of waste collection and transportation. In a stochastic environment, a credibility level should be set in accordance with dispatcher’s risk preference, then make the best use of smart waste bins to choose the optimum WFL between 0.65 and 0.75 to obtain the maximum tightness, minimum unit cost, and appropriate collected waste percentage.

From the perspective of government environment departments, firstly, they’d better introduce some relevant policies to accelerate the application of smart waste bins to raise the efficiency of waste management system. Secondly, they must strengthen people’s awareness of environmental protection, and encourage the enterprise to save energy and reduce carbon emissions.

## 6. Conclusions

With the development of intelligent technology, smart waste bins have been applied in some regions of China, which can rise the efficiency of SWM. Waste collection and transportation is a high-carbon emissions stage in SWM with a stochastic waste generation rate. It is necessary to optimize the waste collection routes while taking into environmental benefits based on the data from smart waste bins. In this paper, based on the application of smart waste bins, a comprehensive CCLCVRP model, with the minimized total cost including vehicle cost and carbon emissions cost, is designed to optimize the wet waste collection and transportation paths. An improved genetic algorithm, PSOSA, is introduced to solve the model. Moreover, the numerical experiments are used to verify the effectiveness of the algorithm. Then a case data is used to validate the established model. The minimum total cost, the tightness, unit cost and total collected waste percentage are calculated respectively as the reference for subsequent experiments with different WFLs and credibility levels. Based on the results, some suggestions are provided for the department of waste management and waste collection and transportation organizations. In future research, the CCLCVRP based on the application of smart waste bins can consider multiple vehicle types and all kind of waste. In addition, real data can be used to get some more realistic and reliable examples.

## Figures and Tables

**Table 1:** Description of symbols.

Notation	Explanation
B	Set of smart waste bins (B=1, 2, ⋯, N)
V	Set of vehicles (V=1, 2,⋯, K)
L	Longest length of each route
$q_{i}$	Initial waste of bin i
$\Delta q_{i}$	Incremental waste of bin i, $\Delta q_{i}\sim N\left(\mu_{i},\sigma_{i}^{2}\right)$
$Q_{0}$	Weight of vehicle itself
$Q_{1}$	Load weight of vehicle
Q	Maximum load capacity of vehicle
$\rho_{0}$	Fuel consumption rate per unit distance while vehicle is empty (L/km)
ρ	Fuel consumption rate per unit distance (L/km)
$\rho^{*}$	Fuel consumption rate per unit distance while vehicle is at full load (L/km)
$\rho_{idel}$	Fuel consumption rate per unit time while vehicle idling
$F_{fuel}$	Total amount of fuel consumption (L/min)
$E_{CO_{2}}$	Total amount of carbon emissions from fuel consumption.
η	Conversion factor value of fuel consumption and carbon dioxide
ε	Cost of per unit carbon emission
$d_{ij}$	Distance between smart waste bin i and j
$t_{i}$	Service time of smart waste bin i
$p_{f}$	Fixed cost of per unit vehicle
$x_{ijk}$	If the vehicle k visits bin j from i, $x_{\mathrm{ijk}}$ *i*s 1. Otherwise, $x_{ijk}$ is 0
p	Price of per unit fuel consumption

**Table 2:** Allocation of smart waste bins.

3	2	1	1	2	3	2	3	1	1
1	2	3	4	5	6	7	8	9	10
0.7358	0.4398	0.6832	0.3605	0.0735	0.0884	0.9307	0.3978	0.3530	0.7460
5	6	9	4	8	2	3	1	10	7
Sub-path1	0	4	10	3	9	0			
Sub-path 2	0	7	2	5	0				
Sub-path 3	0	8	6	1	0				

**Table 3:** Data about the test instances.

Case	Node	Capacity
A-n32-k5	31	100
A-n36-k5	35	100
A-n46-k7	45	100
A-n53-k7	52	100
A-n62-k8	61	100
A-n80-k10	79	100
B-n51-k7	50	100
F-n135-k7	134	2210
P-n76-k5	75	280
P-n101-k4	100	400

**Table 4:** Parameters of PSOSA.

Description	Parameter	Value
Number of the population	$N_{p}$	20
Inertia weight	ω	0.7
Inertia weight damping ratio	ωdamp	0.99
Personal learning coefficient	$c_{1}$	1.5
Global learning coefficient	$c_{2}$	1.5
Evolution terminate generation	$M_{p}$	1000
Initial temperature	$T_{0}$	1000
Cooling coefficient	r	0.9
Final temperature	$T_{end}$	1

**Table 5:** Results of PSO and PSOSA.

Database	PSO			PSOSA		
	Cost	Length	Carbon Emissions	Cost	Length	Carbon Emissions
A-n32-k5	3889	1628	1628	3587	1543	974
A-n36-k5	6005	1623	1127	5256	1454	1005
A-n46-k7	5039	2075	1419	4771	1893	1382
A-n53-k7	6982	2925	2090	3856	2463	1918
A-n62-k8	10,449	3043	2367	9035	2766	2053
A-n80-k10	15,103	4531	2836	9997	4471	2641
B-n51-k7	8552	2864	1905	6203	2648	1697
F-n135-k7	14,645	5946	5876	12,906	5671	4052
P-n76-k5	10,227	2573	1390	9576	2369	1237
P-n101-k4	7091	2865	1762	6739	2680	1628

**Table 6:** Information about the Depot and Smart Waste Bins.

Point	X Coordinate	Y Coordinate	Amount of Waste
Depot	2.3	2.12	—
1	0.98	0.08	376.122
2	3.6	1.05	339.786
3	3.35	2.68	463.5
4	1.92	4.27	548.34
5	2.46	4.55	551.1
6	3.87	1.67	550.002
7	0.74	2.35	361.116
8	2.43	0.01	463.326
9	0.36	1.55	562.482
10	3.94	2.43	336.3
11	1.97	1.31	556.908
12	1.18	3.42	569.934
13	0.4	4.56	365.358
14	0.4	2.85	323.094
15	4.64	1.33	442.266
16	1.02	4.64	550.506
17	4.78	0.32	440.82
18	1.7	3.13	424.128
19	0.3	0.54	450.822
20	1.72	2.58	337.632
21	3.02	4.78	339.684
22	3.06	1.36	561.144
23	2.78	2.63	480.888
24	2.73	3.56	379.59
25	1.01	1.07	559.44
26	3.94	4.13	317.43
27	1.74	0.69	437.328
28	4.86	2.19	516.66
29	0.58	3.88	401.7
30	3.56	3.52	420.366

**Table 7:** Parameters related to the objective function.

Parameters	Value
$p_{f}$	100 CNY
p	8 CNY//L
$\rho_{0}$	0.165 L/km
$\rho^{*}$	0.377 L/km
$\rho_{idel}$	0.05 L/min
η	2.63 kg/L
ε	0.025 CNY/kg

**Table 8:** the Robustness of EVM and CCP.

Results	EVM	CCP (γ)				
		0.6	0.7	0.8	0.9	0.99
Robustness	45%	55%	75%	80%	90%	97%

**Table 9:** Obtained detailed results by applying the WFL concept.

WFL	C	CE	L	I	n	N	Route	TCW	$TCW_{p}$	T	UC
WFL = 0	945	80	91	0%	30	7	(0,11,3,29,4,0)	13,428	100%	96%	0.07035
							(0,23,26,9,5,0)				
							(0,20,17,18,14,8,0)				
							(0,12,24,25,30,0)				
							(0,7,28,10,6,0)				
							(0,16,22,15,2,0)				
							(0,27,19,21,1,13,0)				
WFL = 0.9	350	16	18	63%	9	3	(0,7,1,2,0)	5010	37%	83%	0.06983
							(0,9,4,6,0)				
							(0,3,8,5,0)				
WFL = 0.8	460	20	23	51%	11	4	(0,4,10,5,0)	6007	45%	75%	0.07659
							(0,2,6,0)				
							(0,1,7,9,0)				
							(0,11,3,8,0)				
WFL = 0.75	583	27	31	38%	14	5	(0,6,9,8,0)	7385	55%	74%	0.07889
							(0,5,1,0)				
							(0,10,13,7,0)				
							(0,12,2,3,0)				
							(0,11,14,4,0)				
WFL = 0.7	632	43	47	33%	19	5	(0,18,16,17,5,0)	9550	71%	96%	0.06618
							(0,6,2,3,0)				
							(0,4,9,7,13,0)				
							(0,12,15,1,14,0)				
							(0,19,10,8,11,0)				
WFL = 0.65	728	42	48	23%	20	6	(0,1,15,20,4,0)	9952	74%	83%	0.07312
							(0,8,18,11,9,0)				
							([0,2,16,14,0)				
							(0,5,17,13,6,0)				
							(0,19,3,7,0)				
							(0,10,12,0)				
WFL = 0.6	776	58	67	18%	24	6	(0,23,11,10,17,0)	11,434	85%	95%	0.06791
							(0,6,15,22,20,0)				
							([0,19,9,8,18,0)				
							(0,13,1,16,12,0)				
							(0,14,7,21,3,0)				
							(0,24,5,4,2,0)				
